# A 1,000 Frames/s Programmable Vision Chip with Variable Resolution and Row-Pixel-Mixed Parallel Image Processors

**DOI:** 10.3390/s90805933

**Published:** 2009-07-27

**Authors:** Qingyu Lin, Wei Miao, Wancheng Zhang, Qiuyu Fu, Nanjian Wu

**Affiliations:** State Key Laboratory for Superlattices and Microstructures, Institute of Semiconductors, Chinese Academy of Sciences, Beijing 100083, China; E-Mail: qylin@red.semi.ac.cn (Q.-Y.L.)

**Keywords:** vision chip, image processing, machine vision, mathematical morphology

## Abstract

A programmable vision chip with variable resolution and row-pixel-mixed parallel image processors is presented. The chip consists of a CMOS sensor array, with row-parallel 6-bit Algorithmic ADCs, row-parallel gray-scale image processors, pixel-parallel SIMD Processing Element (PE) array, and instruction controller. The resolution of the image in the chip is variable: high resolution for a focused area and low resolution for general view. It implements gray-scale and binary mathematical morphology algorithms in series to carry out low-level and mid-level image processing and sends out features of the image for various applications. It can perform image processing at over 1,000 frames/s (fps). A prototype chip with 64 × 64 pixels resolution and 6-bit gray-scale image is fabricated in 0.18 μm Standard CMOS process. The area size of chip is 1.5 mm × 3.5 mm. Each pixel size is 9.5 μm × 9.5 μm and each processing element size is 23 μm × 29 μm. The experiment results demonstrate that the chip can perform low-level and mid-level image processing and it can be applied in the real-time vision applications, such as high speed target tracking.

## 1. Introduction

A vision chip integrates a sensor array with parallel processors in one chip and performs real-time parallel low- and mid-level image processing without I/O bottlenecks. It has advantages of compact size, high speed, and low power consumption so that it can be widely applied in many fields such as robotics, industrial automation and target tracking systems [1,2]. One of the challenges in the development of the vision chip is how to take advantages of the parallel-processing performance of vision chip to realize complex real-time algorithms. This paper presents a programmable vision chip with variable resolution and row-pixel-mixed parallel image processors, which can perform such complex algorithms.

Image processing methods can be cataloged into three kinds of levels: low-, mid-, and high-level processing [3]. Low-level processing involves primary operations such as noise cancellation and image enhancement. Mid-level processing involves segmentation, description of regions and classification of objects. High-level processing performs intelligent analysis and cognitive vision. The common features of low- and mid-level image processing include parallel image processing and large amount of processing image data. The vision chip can perform row-parallel and pixel-parallel image processing. Therefore the vision chip is suitable for low- and mid-level image processing tasks.

General-purpose gray-scale image vision chips with analog processing elements (PE) have been reported in [4–8]. These general-purpose vision chips could only handle low-level image processing and the large amount of outputs image data limits their application in real-time image processing tasks. To solve the problem, a general-purpose vision chip is required that could perform low- and mid-level image processing in the chip subsequently. Therefore the ability of the vision chip must be improved and the I/O bottleneck must be overcome by sending out less image feature data.

Some application-specific vision chips performing low- and mid-level image processing were developed [9–12], but these chips were designed for specified applications and with a specified architecture. Another vision chip performing exact dilations was presented in [13], but this one focused on several specific algorithms, such as morphological dilation, multi-scale skeleton and Distance Transform [14]. They were not programmable or feasible for general purpose applications.

We also have developed a vision chip that performs low- and mid-level image processing [15,16]. The chip was based on specified mathematical morphology algorithms for high-speed target tracking. It could only accomplish binary image processing.

In this paper, we present a 1,000 frames/s (fps) programmable vision chip with variable resolution and row-pixel-mixed parallel gray image processors. The chip overcomes the difficulty of the early proposed general-purpose vision chips in the field of real-time machine vision. It consists of *2N* × *2N* CMOS image sensor, *N* row-parallel 6-bit Algorithmic ADCs, *N* gray-scale image processors, and *N* × *N* Single Instruction Multiple Data (SIMD) pixel-parallel PE array. The chip mainly performs low- and mid-level image processing based on *gray-scale* and *binary mathematical morphology* method and outputs image features for high-level image processing. The chip can move the focused area in one image and change image resolution to perform image processing under different environments or application. The chip can implement various complex algorithms for real-time machine-vision applications by software control. The chip has features of high-speed, low power consumption and small pixel element.

The rest of the paper is organized as follows. In Section 2, we describe the architecture and operations of the chip. In Section 3, the implementation of the chip is presented. In Section 4, we give some image processing examples, including a target tracking algorithm using a prototype chip. In Section 5, the performance of the chip is discussed. Finally, we come to the conclusions in Section 6.

## 2. Architecture and Operations of the Chip

### 2.1. Architecture

**Figure 1.**
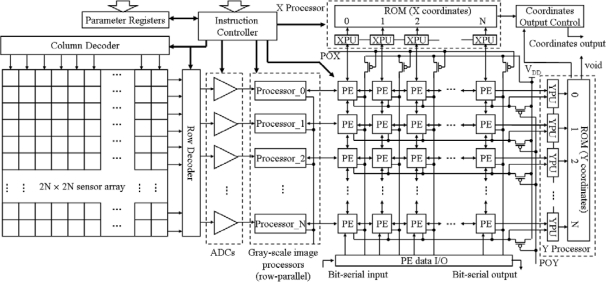
Architecture of the vision chip.

The architecture of the proposed programmable vision chip with variable resolution and row-pixel-mixed parallel gray image processors is shown in Figure 1. The vision chip consists of *2N* × *2N* image sensor, *N* ADCs, *N* gray-scale image processors (row-parallel processors), *N* × *N* PE array, X processor, Y processor, instruction controller, parameters register, and output module.

**Figure 2.**
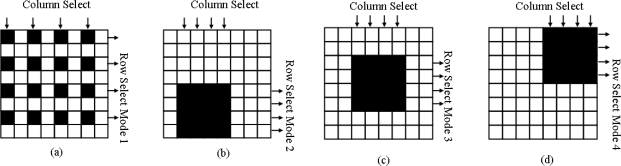
Row Select modes. (a) Row select mode 1, select interleaved rows (b) Row select mode 2, select the 32 rows in bottom of the image, (c) Row select mode 3, select the mid 32 rows (d) Row select mode 4, select the top 32 rows. In mode 2 3 4, any continuous 32 columns can be selected.

The image sensor module consists of *2N* × *2N* 3-transistors photodiode-type active pixel sensor (APS) [17] array, row and column decoder circuits in the periphery of the sensor array. The row decoder is realized by a multiplexer of four inputs and is controlled by the instructor from the parameter register. Figure 2 shows that the decoder can work in four different modes. In each mode the sensor array outputs *N* rows of the whole *2N* × *2N* image into the *N* ADCs module. The column decoder is a common decoder and it is controlled by a Finite State Machine (FSM). By FSM instruction, the column decoder can select *N* columns from the *2N*-column image in column by column or one in every two columns. Therefore an *N* × *N* area selected in the *2N* × *2N* image can be output into the *N* ADCs module. The feature of this image sensor module is that it can emulate the human eye function and focus on a specified area of the image.

The ADCs module consists of *N* row-parallel 6-bit ADCs. Even though a lot of work, such as reported in [18–21], has been done on pixel level ADCs; we still chose the row parallel (named column parallel in some papers) structure [22] for analog to digital converting. The ADC we implemented in the chip is based on an algorithmic approach [23]. It can fit the vision specifications and has the features of smaller chip area and lower power consumption than pixel level ADC. It can convert the signals of *N* pixels in one column simultaneously. The convert period time depends on the gray-scale resolution.

**Figure 3.**
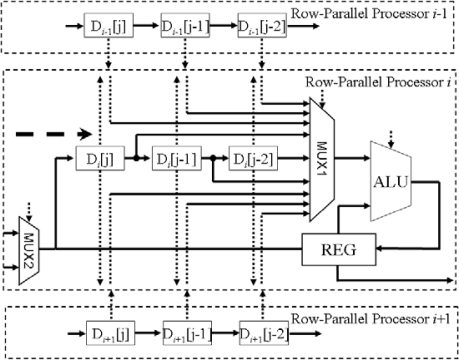
The interconnection between row-parallel processors.

For a binary image, the ADC finishes the converting operation in 2 clock cycles. For a 6-bit gray-scale image, the ADC converts the image in 7 clock cycles. Therefore we can control the quality of the image and the converting time to suit the different vision application. The gray-scale image processors receive the digital image from *N* ADCs or PE array. Each processor consists of three 6-bit pixel-data registers and an 11-bit ALU, as shown in Figure 3.

The ALU in row-parallel processor *i* can access three pixel-data registers D*_i_*[j] D*_i_*[j−1] D*_i_*[j−2] in itself, three pixel-data registers D*_i_*_−1_[j] D*_i_*_−1_[j − 1] D*_i_*_−1_[j − 2] in row-parallel processor *i −* 1 and three pixel-data registers D*_i_*_+ 1_[j] D*_i_*_+ 1_[j − 1] D*_i_*_+ 1_[j − 2] in row-parallel processor *i* + 1. We terminal the boundary of the sensor array with low voltage (logic ‘0’). This boundary condition is required by *mathematical morphology* image processing. The ALU can process the data of 3 × 3 array in the image and perform 8 basic operations including *‘add’ ‘subtract’ ‘minimum’ ‘maximum’ ‘comparison’ ‘equal’ ‘absolution’* and *‘shift’*. These processors would process one column image data each period. The *gray-scale mathematical morphology* algorithms can be executed by combination of those operations repeatedly and successively.

**Figure 4.**
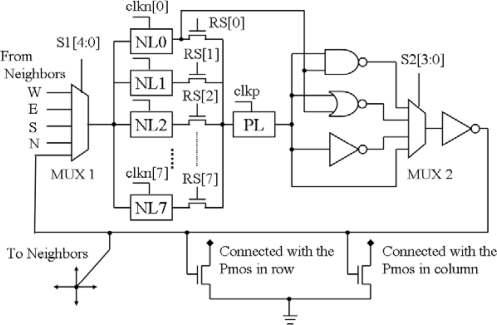
The diagram of PE.

The core module of the chip is an *N* × *N* PE array. The PE diagram is given in the Figure 4. It consists of nine D-latches, two Multiplexers, an AND gate, an OR gate, an inverter, and eight switches. One PE is connected directly with its four nearest neighborhood PEs. By selecting the MUX1, we can choose one signal as input of the PE, which comes from the neighbor PE or the output of itself. When the switch (RS[i]) was turned on, the negative latch (NL[i]) and positive latch (PL) constitute a D-flop. There are 8 D-flops that can store 8-bit data in one PE. The clocks (clkn[0-7]) of the eight NL[i] (i = 0, 1,…,7) are controlled independently by the instruction controller. PE can perform logical operations between the data in one of NL[1–7] and in NL[0] when we switch on one of RS[1–7] correspondingly. MUX2 is used to select one of the different operations: AND, OR, Invert, and Keeping Data.

There are an X Processor, a Y Processor, a PE data I/O module, and 2*N* PMOS transistors in the periphery of the PE array. One PMOS transistor and *N* NMOS transistors in the PE array constitute an *N*-input pseudo-NMOS NOR gate. The *N* transistors are contained respectively in *N* PEs. There are *N* rows *N*-input pseudo-NMOS NOR gates and *N* column N-input pseudo-NMOS NOR gates in the PE array. The outputs of these *N*-input pseudo-NMOS NOR gates were connected with the X processor and the Y processor.

The X processor contains *N* X Processor Units (XPU) and *N* 5-bit ROM that stores the X coordinates. The X processor receives the projection of the binary image, which is stored in all NL[i] of each PE, on the X-axis. The positions of the left edge and the right edge of the projection are found by XPUs. The X coordinates of the positions are obtained by checking the ROM.

The Y processor contains *N* Y Processor Units (YPU) and *N* 5-bit ROM that stores the Y coordinates. Its inputs are either the outputs of the *N*-input pseudo-NMOS NOR gates in rows or parallel shift-out data of the binary image in PE array. The Y processor not only gives the Y coordinates of the top edge and the bottom edge of the projection of the image on the Y-axis, but also obtains the Y coordinates of the activated pixels in a column of the image in sequence. The X coordinates and the Y coordinates from the X processor and the Y processor are transferred to the coordinates’ output module. The instruction controller connects with the outside circuit. It receives all of algorithms instructions and sends them to other modules. Parameters registers store the initial parameter for each module. The output module receives the X coordinates and the Y coordinates from the X processor and the Y processor and output them in series.

### 2.2. Operations

The proposed programmable vision chip can perform image acquisition, low and mid-level image processing, and fast output of features of images in a designed procedure. The low- and mid-level image processing is mainly carried out by the *Mathematical Morphology* method [3]. The chip can process gray-scale image and binary image in series. First the gray-scale image processors use the *gray-scale mathematical morphology* to perform the gray-scale image processing in the row parallel fashion. After some low-/mid-level image processing is finished and the precise skeleton of some objects in the image is extracted, the chip binaries the gray-scale image and uses *binary mathematical morphology* to process the image in pixel parallel fashion subsequently. The speed of the pixel parallel processing is *N* times faster than row parallel processing. The data of binary image is less than gray-scale. The sequence of the gray-scale image and binary image processing can avoid a trade-off between the performance time of each frame and the accuracy of the output data so that we can carry out the image processing quickly and obtain the useful feature information from the image.

#### 2.2.1. Binary Mathematical Morphology

Morphological *Morphology* is an advanced image processing method that is based on the basic logical operations. The method not only performs low-level image processing such as morphological filtering, but also carries out mid-level image processing, such as extracting image objects or features.

The operations in the method can be described by set theory. The two fundamental operations are *erosion* and *dilation*. If *A* and *B* are two images, the *erosion* of *A* by *B*, denoted *A Θ B*, is defined as:

(1) $$A\;\Theta \;B=\underset{b\in B}{\cap }{\left(A\right)}_{-b}$$

and the *dilation* of *A* by *B*, denoted *A ⊕ B*, is defined as:

(2) $$A\;⊕\;B=\underset{b\in B}{\cup }{\left(A\right)}_{-b}$$

where the denotation *(A)_b_* is the *translation* or *shift* of the image *A* by point *b = (x, y)*, and is defined as:

(3) $${\left(A\right)}_{b}\;=\;\left\{c|\;=a+b,\;\;a\in A\right\}$$

**Figure 5.**
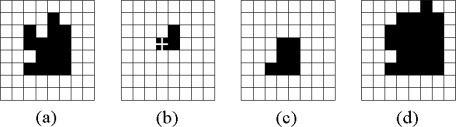
Examples for *erosion* and *dilation* in *mathematical morphology* operations. (a) An image denoted *A*. (b) An image denoted *B*, in which the cross shows where the origin is. (c) The result of *A Θ B*. (d) The result of *A ⊕ B*.

Usually the image *B* is regarded as a structuring element. Examples of *erosion* and *dilation* are given in Figure 5. We assume that the black box represents the activated pixel and its value is logic ‘1’ in a binary image. Figures 5(a) and 5(b) show the original image and the structuring element, respectively. The results of the *erosion* and *dilation* operations are shown in Figures 5(c) and 5(d). Other operations, such as *opening*, *closing*, and *hit-or-miss transform*, are realized by combinations of *erosion*, *dilation,* and logical operations.

*Erosion* and *dilation* are constituted by a serial of logic operations including *shift*, *OR* and *AND*. This programmable vision chip efficiently realizes *erosion* and *dilation* by pipelining operations of *Shift* and *OR* or *AND* during the registers in the PE. The procedure is rather regular regardless of the structuring element. The number of clock cycles used to perform *erosion* or *dilation* in the chip can be estimated as *2M*, where *M* is the number of the activated pixels in the structuring element. All of the operations can be easily implemented by the chip. With a function that detects a *void image* which is defined as an image without activated pixel, advanced algorithms of *mathematical morphology* such as *region growing* and *convex hull* can be implemented in the chip.

#### 2.2.2. Gray-Scale Mathematical Morphology

*Gray-scale mathematical morphology* is extended from *mathematical morphology*. We assume that *f(x, y)* is the input image and *b(x, y)* is a structuring element, itself a sub-image function.

The *gray-scale erosion*, denoted *f Θ b*, is defined as:

(4) $$(f\Theta b)\;(s,t)=\text{min}\left\{f(s+x,t+y)-b(x,y)|(s+x),(t+y)\in D_{f};(x,y)\in D_{b}\right\}$$

and the *gray-scale dilation,* denoted *f ⊕ b*, is defined as:

(5) $$(f⊕b)\;(s,t)=\text{min}\left\{f(s-x,t-y)+b(x,y)|(s-x),(t-y)\in D_{f};(x,y)\in D_{b}\right\}$$

where *D_f_* and *D_b_* are the domains of *f* and *b*, respectively. The expressions for *opening* and *closing* operations of gray-scale image have the same form as their binary counterparts. All of these operations are implemented by the row-parallel processors: the gray-scale image processors. The gray-scale image processors process the gray-scale image in row-parallel and output the data into PE array column by column. Two important operations: *opening* and *closing*, can be realized by the combination of the basic operations, *gray-scale erosion* and *gray-scale dilation*.

The opening of image *f* by sub-image (structuring element) *b*, denoted *f ○ b*, is:

(6) f∘b=(fΘb) ⊕b

Similarly, the closing of *f* by *b*, denoted *f • b*, is:

(7) f•b=(f⊕b) Θb

Most of the gray-scale morphology operations, such as morphological *smoothing gradient* and *top-hat transformation*, can be realized by combining the *gray-scale erosion* and *gray-scale dilation*. This programmable vision chip implements the gray-scale image processing first after the image is captured. The gray-scale morphological operations are a powerful set of tools for extracting features of interesting objects in an image and recognizing them. Then it converts the gray-scale image to binary image and use *binary mathematical morphology* for subsequent processing, for example *Thinning* or *Skeletons* are the means to get the main part of target object. Table 1 shows the operation time performance of *binary* and *gray-scale mathematical morphology* on the structure of our chip.

#### 2.2.3. Detecting a Void Image

**Figure 6.**
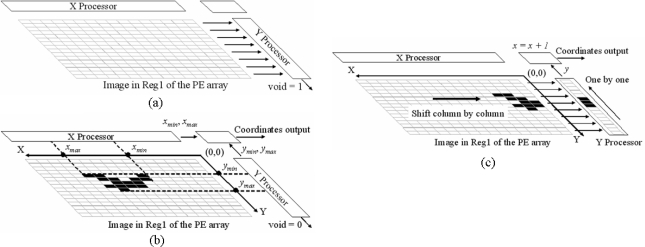
(a) Diagram for detecting a *void image* (Void = 1). (b) Extracting the range and the center of a region. (c) Diagram of extracting the coordinates (*x, y*) of activated pixels in an image.

A very useful function of the chip is to detect whether an image is a *void image*. After *subtraction* is performed between two images, whether the two images are equal is known by detecting if the result image is a *void image*. In many iterative algorithms the instruction controller usually terminates the iterative process by detecting a *void image*. The function of *detecting a void image* can be realized by the NOR gates and the Y processor. If the Y processor detects *a void image*, it will output logic ‘1’ at the port ‘void’. An example is shown in Figure 6(a).

#### 2.2.4. Extracting the Range and the Center of a Region

The architecture of our chip is very efficient for getting the range and the center of the only one region in an image. If there are several regions in the image, it is required to separate them first. Figure 6(b) shows the global operation on the chip. The image in one register array projected onto the X-axis and the Y-axis by the operation of the *2N* pseudo-NMOS NOR gates. The coordinates of the right edge *x_min_* and the left edge *x_max_* of the projection of the region on the X-axis are extracted in the X processor. The coordinates of the top edge *y_min_* and the bottom edge *y_max_* of the projection of the region on the Y-axis are extracted in the Y processor. The four edges indicate the range of the region and are sent to the coordinates output control module, where the center *P_c_(x_c_, y_c_)* of the region is obtained as:

(8) $$P_{c}\left(x_{c},\;y_{c}\right)=P_{c}\left(\left(x_{\text{min}}+x_{\text{max}}\right)/2,\;\left(y_{\text{min}}+y_{\text{max}}\right)/2\right)$$

It needs only eight clock cycles to calculate the center and the range of a region. The frequency of the clock is determined by the NOR gates and the circuits in the X processor and the Y processor.

In some applications, such as target tracking, it usually needs to obtain a point to represent the position of an object. Comparing with extraction operation of the centroid by global summation that is used in other papers [11], the center of the target can be obtained by our vision chip easily and quickly.

#### 2.2.5. Extracting the Coordinates of Activated Pixels

The results of mid-level image processing are that some image features that mostly appear as points, boundaries, edges or skeletons of the objects in the image. It is required to quickly send these features in certain format out of chip to perform further processing. The chip can extract coordinates of activated pixels in the image. Therefore those features in the image can be outputted as the coordinates of activated pixels so that the information transfer is fast without I/O bottleneck from the chip to other digital processors. Another reason of output features in coordinates is that coordinates are easy to be further handled. Many descriptors or representations such as area, curvature, and chain code can be directly obtained from coordinates.

The procedure that the chip obtains the coordinates of activated pixels in the image in one register array column by column is given in Figure 6(c). First, the data from the first column of the image is transferred into the Y processor. Then the Y processor searches the activated pixels in the column one by one from the bottom to the top in order and at the same time generates the Y coordinates of the activated pixels. Then, after all activated pixels in the column are generated; the PE array sends the image data of the next column to the Y processor. The Y processor begins to generate the coordinates of activated pixels in the new column. The above process is repeated until the Y coordinates of the activated pixels in the last column of the image is generated. On the other hand, the X coordinates are simply generated by a column counter in the coordinates’ output control module.

A vision chip reported in [10] has similar function of searching activated pixels and extracting their coordinates. It used row-parallel-search architecture with 432 MHz clock frequency. Its parallel search and high-frequency cost large area and power consumption. Additionally, the processing speed of the whole system is seriously limited by the digital data readout. Therefore, before coordinates are send out, buffers are used to store them so that the chip area increases. In comparison with the chip, the architecture of our chip is more reasonable by only placing the search circuits in the Y processor to make the speed of extracting and sending the coordinates compatible. In such way, the clock frequency in the PE array can be much lower than that in the Y processor, no circuits for searching activated pixels exist in the PE array, and no output buffer is used. As the results, area and power are saved much.

## 3. VLSI Circuits Design

This vision chip is implemented in 0.18 μm CMOS technology with 3.3 V and 1.8 V multiple voltage supplies. The design of main circuit blocks is demonstrated as follows.

### 3.1. CMOS Image Sensor

A 3-transistors photodiode-type APS [17] in standard salicide CMOS process is used in the chip. The APS CMOS image sensor has been widely discussed in [24]. According to the requirement of 1,000 pfs performance, the integral time of photodiode has to be less than 1 ms for each frame. It needs a high sensitivity photodiode in the sensor array, so that an N-well/P-sub SAB diode without salicide is used as photodiode. SAB is the mask layer of salicide block mask, which is used only in standard salicide CMOS process for blocking salicide formation. Using the photodiode, we gain short integral time and high relative spectral quantum efficiency. The test result and explanation of N-well/P-sub SAB diode without salicide has been presented in our previous work [16].

### 3.2. Row-Parallel 6-bit Algorithmic ADCs

**Figure 7.**
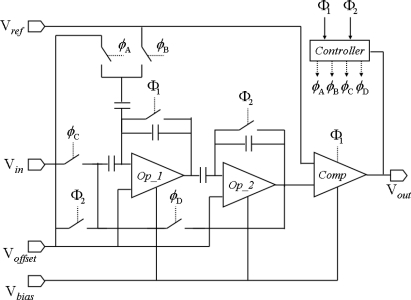
The diagram of algorithmic ADC.

The algorithmic ADC has been widely used in CMOS image sensors for many years [23]. In the vision chip, we chose a traditional structure of algorithmic ADC. The diagram of the ADC shows in Figure 7. V*_in_* is the output signal of one pixel in the sensor array. V*_bias_* is the circuit bias voltage. V*_ref_* and V*_offset_* are two off-chip reference voltages for analog-to-digital converting. *Φ*_1_ and *Φ*_2_ are non-overlapping two-phase clocks. *Φ*_A_ *Φ*_B_ *Φ*_C_ *Φ*_D_ are switch signals derived from *Φ*_1_ and *Φ*_2_. The sample signal is multiplied by 2 in the operational amplifier *‘Op_1’* and hold in *‘Op_2’*. Then the output voltage of *‘Op_2’* compares with a reference voltage in comparator ‘*Comp’*. After comparison the digital result outputs 1 bit by 1 clock cycle of *Φ*_1_ or *Φ*_2_. At the next clock, the output voltage of *‘Op_2’* recycles to the input of ADC for next bit of the digital output. An analog signal converting into a 6-bit digital signal takes seven clock cycle, one for sampling and six for 6-bit output.

### 3.3. Row-Parallel Processors

**Figure 8.**
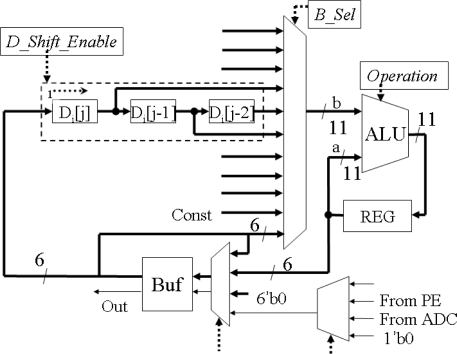
The diagram of row-parallel processor architecture.

The row-parallel processor is designed for calculating sum, subtraction and comparison of two 6-bit data. The diagram is shown in Figure 8. The ‘Buf’ converts the serial input data to the parallel data. *‘D_Shift_Enable’* controls the data to transfer column by column. *‘B_Sel’* switches the input of the ALU. Because the maximum of the sum of nine 6-bit data is less than 11-bit, so the data width of ALU is designed as 11-bit. It composed of eleven single-bit ALUs. The operating instruction, *‘Operation’*, comes from off-chip circuits.

### 3.4. Search Chain in X Processors and Y Processors

**Figure 9.**
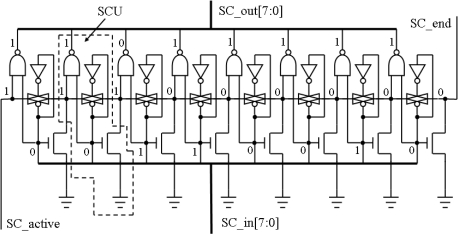
Schematic diagram of a search chain with 8 Search Chain Units (SCU).

The search chain can perform a function that finds out the first logic ‘1’ in a serial of bits along with certain direction. The length *L* of the search chain is defined as the number of bits being searched in the chain. An example of the search chain that has *L = 8* is given in Figure 9. The search chain consists of 8 search chain units (SCU). It finds out the position of the first logic ‘1’ from the left to the right in the 8-bit parallel inputs ‘SC_in’. At the position of the first logic ‘1’, the corresponding bit in parallel outputs ‘SC_out’ will become logic ‘0’, and other bits are all logic ‘1’. The input signal ‘SC_active’ controls the operation of the search chain. The search chain operates only when the ‘SC_active’ is set to high. Another output signal ‘SC_end’ comes from the end of the search chain. If ‘SC_active’ is high, ‘SC_end’ will be high only when all parallel-input bits of the search chain are logic ‘0’. ‘SC_end’ is quite useful in some operations. For example, the output port ‘void’ of the Y processor is directly connected with ‘SC_end’. Search circuits realized by dynamic logic with similar function were reported in [9] and [10]. Comparing with the dynamic logic circuit, the static logic circuit of our search chain has advantages of easy implementation and tolerance to noise.

The search time is principally determined by the delay of the transmission gates and is proportional to the length *L* of the search chain. The longest delay is 12.12 ns if *L* is 128 and a buffer is inserted between every three transmission gates. The search time is much less than that of the search circuits in [9] and [10]. The longest search time is 71 ns for 128 pixels per row in [9] and 30 ns in [10].

#### 3.4.1. XPUs in the X Processor

**Figure 10.**
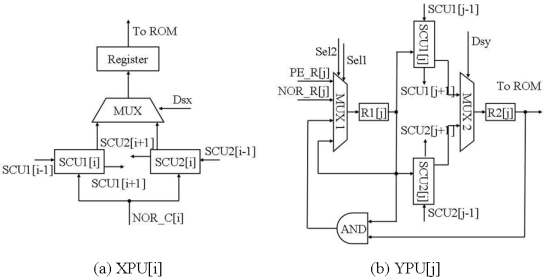
(a) Schematic diagram of XPU[i]. (b) Schematic diagram of YPU[j].

The X processor contains *N* XPUs. The diagram of the *i*-th XPU, *i = 1, 2, 3,…,N* is shown in Figure 10(a). It consists of two search chain units SCU1[*i*] and SCU2[*i*] which belong to two search chains with opposite search direction. The input NOR_C[*i*] of the two SCUs is the output of the NOR gate located in the *i*-th column of the PE array. A multiplexer selects the outputs of the two SCUs, and the outputs are sent to the ROM that stores X coordinates.

#### 3.4.2. YPUs in the Y Processor

The diagram of the *j*-th YPU, *j = 1, 2, 3,…,N* is given in Figure 10(b). Like the *i*-th XPU, it also consists of two search chain units SCU1[*j*] and SCU2[*j*] which belong to two search chains with opposite search direction. It has an additional input PE_R[*j*] that is the data from the *j*-th row of the PE array except the NOR_R[*j*], which is the output of the NOR gate in the *j*-th row. If the bit in the register R1[*j*] of the *j*-th YPU is the first logic 1 that is found by the search chain 2 formed by SCU2[*j*], the bit is set to 0 by the path where the AND gate locates. Hence the search chain 2 can continue to find out the next bit that has the value of 1. When the chip performs the operation of *extracting coordinates of activated pixels*, the search chain 2 works. During the process, Sel1 will equal to the signal ‘SC_end’ of search chain 2. This makes the process automatically continue after initialization of the control signals in the Y processor.

## 4. Chip Implementation and Experiments

**Figure 11.**
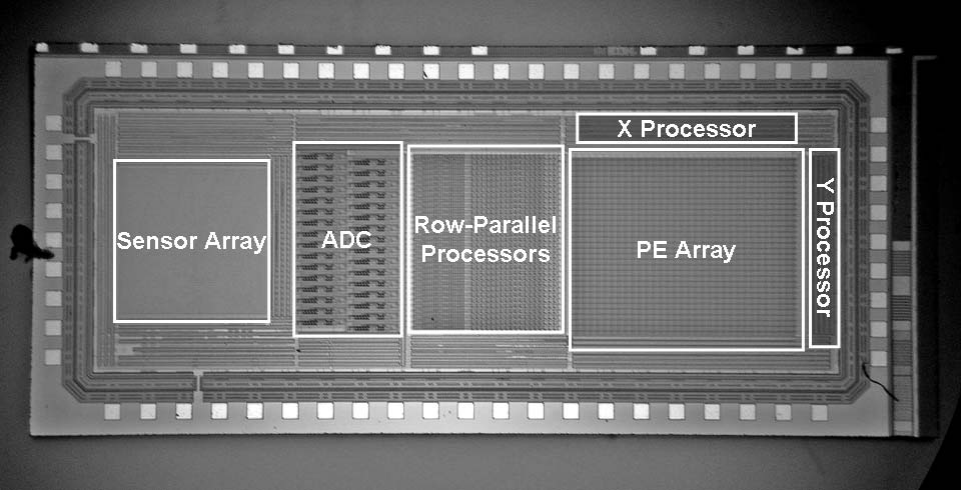
Microphotograph of the prototype chip.

The vision chip was designed and fabricated by 0.18 μm Standard CMOS process. The microphotograph of the chip is given in Figure 11. Table 2 lists the chip's specifications. Experimental results and performance of the vision chip are given below.

### 4.1. Experiment in Gray-Scale Mathematical Morphology

**Figure 12.**
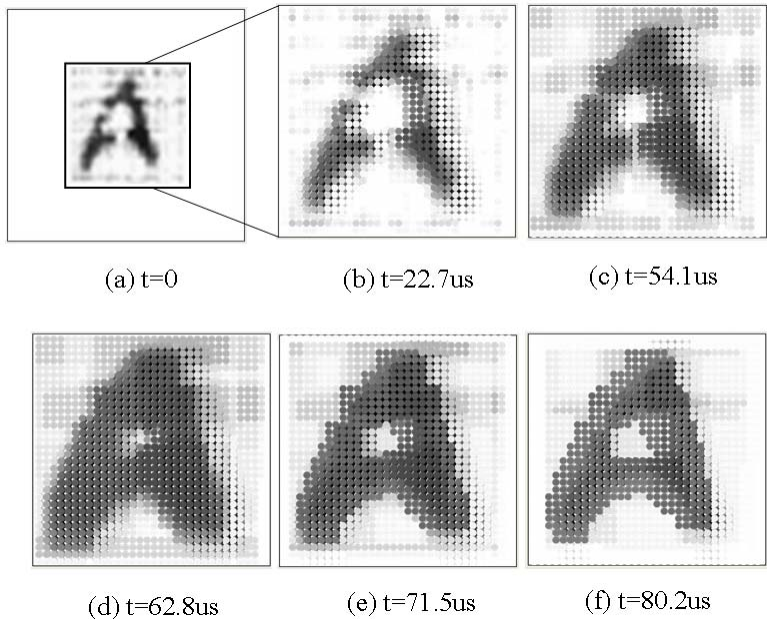
An example of the algorithms using *gray-scale mathematical morphology* performed in the prototype chip. (a) A hand-written English letter ‘A’. (b) A selected 32 × 32 pixels zoom as the focus image. (c), (d) the gray-scale image after two *opening* operations. (e), (f) the gray-scale image after two *opening* and two *closing* operations.

In this experiment, we used *gray-scale mathematical morphology* to smooth the image and fix some disconnect in the image. We used a hand-written English letter ‘A’ as the source image, as shown in Figure 12(a). First the CMOS image sensor obtained an original 64 × 64 pixels image and selected a 32 × 32 pixels zoom as the focused image area, and sends it to ADCs, as shown in Figure 12(b). The row-parallel processors performed the *opening* and *closing* operation on the image. The template image data were stored in PE array. Figures 12(c), (d), (e) and (f) shows the results after two *opening* and two *closing* operations, respectively. Thus the illegible ‘A’ was clearer than the original image. The gray-scale image processing took 80.2 us (3,208 cycles on a 40 MHz clock).

### 4.2. Experiment in Binary Mathematical Morphology

**Figure 13.**
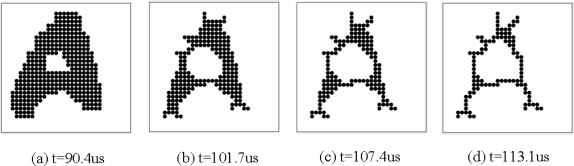
An example of the algorithms using *binary mathematical morphology* performed in the prototype chip. (a) Binary image of the converted illegible letter ‘A’. (b), (c) Performing *thinning* operations. (d) A skeleton of the letter ‘A’.

After the row-parallel image processing, the illegible letter ‘A’ gray-scale image was converted into a binary one in the row-parallel processors. Figure 13(a) shows a binary image of the converted illegible letter ‘A’. Then the binary image processing was performed by the PE array in pixel-parallel binary *mathematical morphology* fashion. Figure 13(b–d) shows the images after *thinning* operations were performed. Thus we got a skeleton of the letter ‘A’. It took 22.7 us (908 cycles of a 40 MHz clock) for this whole binary image processing and was one fourth of the total performance time.

### 4.3. Target Tracking

**Figure 14.**
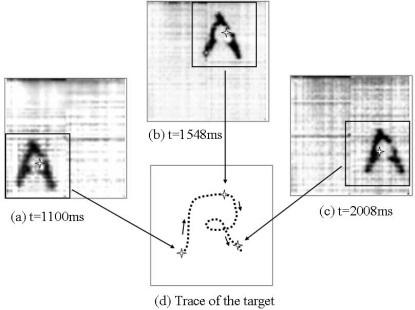
An example of the experiment of target tracking. 3 samples of image during the tracking process, (a) t = 1,100 ms, (b) t = 1,548 ms and (c) t = 2,008 ms. (d) The trace of a moving letter ‘A’.

Here we give out an application example of a target tracking. After binary *mathematical morphology* image processing, we obtained a target in a selected zoom area. The X Processor and Y Processor gave the X and Y coordinates of the periphery edges of the projection of the target respectively, and calculated the centroid of the target. The whole processing time includes gray-scale image processing, gray-scale to binary converting, binary image processing and coordinates extracting, and is less than 1ms (40,000 cycles of a 40 MHz clock), so that this chip can perform 1,000 fps target tracking application. It also indicates that in 1ms we can perform more complex algorithm, such as *Top-hat transformation*, *textural segmentation* or *granulometry*, because of the enough cycles to utilities. The limitation of the capture frames per second is not the algorithms or circuit but the sensitivity of CMOS image sensors. In this experiment, we used the skeleton of letter ‘A’ for calculation the coordinates. It is better than using the original image directly, because after the algorithms region filling and an *erosion* operation, the results will be a small object stood for the main part of the object. Figure 14(d) shows the trace of a moving letter ‘A’. Figurea 14(a), (b) and (c) are three samples of image during the tracking process, when the time is 1,100 ms, 1,548 ms and 2,008 ms, respectively. The chip can track the moving target and provide its centroid coordinates.

## 5. Discussion of Performance

The chip area is mostly determined by the *N × N* PE array that increases with the square of *N*. Therefore, the area of the PE is a very important parameter. The area of each PE in the prototype chip is 23 × 29 μm^2^. It is large potential to reduce the area of the PE if reducing some performance is acceptable for specified applications. In contrast with other works [13,25–21], our vision chip gets more efficient performance and programmability. That is benefit from the flexibility of gray-scale mathematical morphology. Table 3 shows the comparison.

In our vision chip, 6-bit ADC has been implemented. If a high quality image is required, we can design 8-bit or 10-bit ADCs in the chip, but it will use more chip area and more power consumption. The choice of 6-bit ADC is a trade-off between the quality and the cost. For most applications of *gray-scale mathematical morphology*, 6-bit image is enough.

Considering a more practical vision chip with more pixels derived from this one, for example a vision chip with a 256 × 256 pixels PE array, we can still run it at 1,000 fps on a 40 MHz clock. Assuming we perform the same algorithm in the 256 × 256 pixels vision chip as presented in this paper, then the duration of gray-scale image processing in row-parallel is proportional to the number of columns *O*(*N*), and the performing time of binary image processing in pixel-parallel is irrelevant to the number of pixels *O*(1). Therefore we can estimate as follows: the total available time for 1 frame is 1 ms or 40,000 cycles of a 40 MHz clock; the gray-scale image processing takes 8 × 80.2 μs (25,664 cycles); the binary image processing takes 22.7 μs (908 cycles of a 40 MHz clock); the total time is 664.3 μs (26,572 cycles), which is still less than 1 ms.

## 6. Conclusions

A programmable vision chip with variable resolution and row-pixel-mixed parallel image processors was presented. The chip consists of a CMOS sensor array with row-parallel 6-bit Algorithmic ADCs, row-parallel gray-scale image processors, pixel-parallel SIMD PE array, and instruction controller. The chip can change its resolution: high resolution for focused area and low resolution for general view, and perform image processing at an over 1,000 fps rate. The chip architecture supports both gray-scale and binary *mathematical morphology* operations in row- and pixel-parallel fashions. It can carry out low-level and mid-level image processing and sends out features of the image for various applications. A 40 MHz prototype chip with 64 × 64 pixels resolution and 6-bit gray image was fabricated in 0.18 μm Standard CMOS process. The chip’s area was 1.5 mm × 3.5 mm. Each pixel size was 9.5 μm × 9.5 μm and each processing element size is 23 μm × 29 μm. The experiment results demonstrated that it can perform low- and mid-level image processing and be applied in the real-time vision applications, such as high speed target tracking. The chip is supplied by 3.3V and 1.8V multiple voltages. Its power consumption is 82.5 mW (@ 1,000 fps & 40 MHz). It is anticipated that it will find wide applications of real-time vision, such as medical inspection, automatic, robotics, and industrial control systems.

## Figures and Tables

**Table 1.** Time performance.

**Operations**	**Clock cycles**
Load from ADC	≈8 × N
Shift 1 pixel	1
Copy	1
AND/OR/NOT	1
Binary Erosion / Dilation	≈M^(1)^
Gray-scale Erosion / Dilation	≈M × N^(2)^
Detecting a void image	2
Extracting the Range and the center	8
Extracting coordinates of activated pixels	≈2K^(3)^
Bit-serial binary image input/output	≈N × N

(1) M is the number of active pixels in a structure element.

(2) N × N is the pixels of an image.

(3) K is the number of active pixels in a binary image.

**Table 2.** Chip specifications.

**Parameter**	**Value**
Technology	0.18 μm 1P6M CMOS Std.
Chip Size (pad incl.)	3.5 mm × 1.5 mm
Array Size	64 × 64 pixels for Sensor Array
	32 × 32 pixels for PE Array
Pixel Size	9.5 μm × 9.5 μm for Sensor
	23 μm × 29 μm for PE
Number of trans/pixel	3 trans in Sensor pixel
	85 trans in PE pixel
Fill Factor	58%
Clock frequency	40 MHz
Power supply and consumption	1.8 V & 3.3V
	82.5mW (@, 1,000 fps)

**Table 3.** Comparison.

**Reference**	**Our chip**	**[13]**	**[25]**	**[26]**	**[27]**
Photosensors	Yes	No	Yes	Yes	Yes
Technology	0.18 μm 1P6M	FPGA	0.35 μm 1P5M	0.25 μm	0.6 μm 2P3M
PE area (μm^2^)	23 × 29	68 LE^*^	75.5 × 73.3	83 × 45	20 × 20
Stored bits per PE	8	N/A	32	4	N/A
PE Array	32 × 32	12 × 12	128 × 128	9 × 9	512 × 512
Image processing	6-Bit Gray	9-Bit Gray	8-Bit Gray	4-Bit Gray	Analog
Control Style	SIMD	Regular	SIMD/CNN	Complicated	Regular
Global features	Specific periphery	N/A	Non-specific Periphery	No exportation	Specific periphery
Programmability	High	Specific	High	Moderate	Low

* the chip was implemented in FPGA, therefore the area account by LE is given.
